# Iodine Supplementation in Mildly Iodine-Deficient Pregnant Women Does Not Improve Maternal Thyroid Function or Child Development: A Secondary Analysis of a Randomized Controlled Trial

**DOI:** 10.3389/fendo.2020.572984

**Published:** 2020-10-06

**Authors:** Nicole J. E. Verhagen, Sueppong Gowachirapant, Pattanee Winichagoon, Maria Andersson, Alida Melse-Boonstra, Michael B. Zimmermann

**Affiliations:** ^1^Division of Human Nutrition, Wageningen University and Research, Wageningen, Netherlands; ^2^Institute of Nutrition, Mahidol University, Nakhon Pathom, Thailand; ^3^Division of Gastroenterology and Nutrition, Children's Research Centre, University Children's Hospital Zurich, Zurich, Switzerland; ^4^Department of Health Sciences and Technology, ETH Zurich, Zurich, Switzerland

**Keywords:** iodine deficiency, supplementation, pregnancy, brain development, cognition, infants, children, thyroid

## Abstract

**Background:** Iodine deficiency during pregnancy may be associated with lower offspring IQ, but there are few data on the safety and efficacy of maternal iodine supplementation on child development. In a previously reported multi-center randomized trial conducted in Thailand and India, we assessed the effect of iodine supplementation in mildly iodine-deficient pregnant women on offspring development. In this secondary analysis of that trial, we report data only from the Thai pregnant women in the study, who were more iodine deficient at entry.

**Methods:** Pregnant women in Bangkok, Thailand, were randomized to receive daily 200 μg oral iodine or placebo until delivery. We assessed thyroid size and thyroid function during pregnancy and cognitive and motor development at ages 1, 2, and 5.7 years. The trial was registered at www.clinicaltrials.gov/NCT00791466.

**Findings:** Women (*n* = 514) entered the trial between November 2008 and March 2011 at a mean ± SD gestational age of 11 ± 2.8 weeks; their median (IQR) UIC was 112 (75, 170) μg/L. Mean compliance with supplementation was 88%. We assessed 397 mothers in the 3rd trimester, 231 infants at age 2 y, and 157 children at mean age 5.7 y. During pregnancy, there was a slightly greater decrease in free and total thyroxine concentrations in the iodine group (*p* < 0.05). At age 2 years, the iodine group had borderline lower scores for combined fine and gross motor function (*p* = 0.05), but there were no other significant differences in development. At 5.7 years, there were no significant group differences in child development.

**Conclusion:** Daily iodine supplementation in mildly iodine deficient pregnant women was associated with small negative effects on maternal thyroxine concentrations, but did not affect child development. The safety and efficacy of iodine supplementation in mildly-iodine deficient pregnant women needs to be evaluated further in large randomized controlled trials.

## Background

Adequate iodine intake is important throughout the life cycle (1), but is particularly critical during pregnancy, as iodine is an essential component of thyroid hormones that are needed for normal fetal development (2, 3). Pregnancy sharply increases the requirement for iodine, and WHO recommends an iodine intake of 250 μg/d for pregnant women, compared to 150 μg/d for non-pregnant women (4). Thus, pregnant women are particularly vulnerable to iodine deficiency (ID) when dietary iodine supply is low. The WHO suggests a median (m) urinary iodine concentration (UIC) < 150 μg/L in a population of pregnant women indicates ID (4).

In regions of severe iodine deficiency (a mUIC in pregnant women of <50 μg/L) and endemic goiter, iodine supplementation in pregnancy improves maternal thyroid status and child neurodevelopment (2). However, whether mild-to-moderate maternal ID (a mUIC of 50–149 μg/L) increases risk for maternal thyroid disorders or low IQ in the offspring is unclear (2). A recent systematic review (5) that examined the effects of iodine supplementation in pregnant women with mild-to-moderate ID (defined as a baseline mUIC of 50–149 μg/L) concluded there is little evidence to support recommendations for iodine supplementation in pregnancy in areas of mild deficiency (6, 7). Iodine supplementation may not be without risk: abrupt increases in iodine intake can impair thyroid function and the fetal thyroid appears particularly vulnerable to this effect (8). A large cross-sectional study in Chinese pregnant women reported a mUIC > 250 μg/L increased risk for mild maternal hypothyroidism (9) and two cohort studies assessing the effect of iodine supplementation in European pregnant women have reported supplementation had negative effects on infant psychomotor development (10–12).

As highlighted by Dineva et al. (5), the best evidence to date on this issue comes from our previous randomized controlled trial (RCT) in which pregnant women in India and Thailand (*n* = 832) were randomized to receive daily 200 μg oral iodine or placebo until term (13). Iodine supplementation had no significant effect on child neurodevelopment measured at mean age 5.4 years (13). A limitation of this study was that, although overall the baseline mUIC indicated mild iodine deficiency, the mUIC at the 2 study sites differed: in Thailand, the mUIC was 112 μg/L, while in India, the mUIC was 188 μg/L. Thus, many pregnant women enrolled in India may have been iodine sufficient at baseline. Therefore, in this secondary analysis, we have included data from only the Thai cohort, who were more clearly iodine deficient at baseline.

## Methods

The study was a randomized, placebo-controlled, double-blind intervention trial; the detailed methods have been described previously (13). The study site was Ramathibodi Hospital of Mahidol University in Bangkok, Thailand. At the time of our intervention, household coverage with adequately iodized salt was ≈60% in Thailand, and school-aged children were iodine sufficient based on their median UIC (13). Ethical review boards at Ramathibodi Hospital and at Wageningen University & Research, the Netherlands, approved the study in two steps: the first approval was for studies to 2 years postpartum and the second follow-on approval to 5–6 years postpartum (13). All participating women gave written informed consent, and the study was registered at ClinicalTrials.gov, NCT00791466.

Inclusion criteria were: (1) singleton pregnancy; (2) age 18–40 years; (3) gestational age ≤14 weeks; (4) not lactating; (5) generally healthy; (6) no use of iodine containing supplements. We excluded women with a TSH >6 mIU/L at screening and referred them for treatment. We randomized women, stratified by site, to receive 200 μg iodine given as potassium iodide tablets (Merck, Darmstadt, Germany) or an identical placebo tablet (Merck, Darmstadt, Germany) taken daily until delivery. We gave out tablets at monthly visits and assessed compliance by tablet counting and assessed side effects using a questionnaire (13). At baseline and during the 2nd (20–24 weeks) and 3rd (30–33 weeks) trimester, we measured maternal weight and height, collected venipuncture blood for measurement of thyroid functions, collected a spot urine sample for measurement of UIC; and measured thyroid volume by using ultrasound.

When children were 1, 2, and between 5 and 6 years, we measured height and weight, obtained a spot urine sample and a fingerprick blood sample which was spotted onto filter paper. At 1 and 2 years postpartum, we administered the Bayley Scales of Infant Development (BSID)-III to obtain scores for cognitive, language and motor development (13). We used raw scores as age-specific norms were not available for the Thai population. The BSID-III raw scores were adjusted for infant's gestational age at birth. In children at 5–6 years, to assess verbal, performance and full-scale IQ, we administered the Wechsler Preschool and Primary Scale of Intelligence (WPPSI)-III (13). To assess executive functions, we used the Behavior Rating Inventory of Executive Function (BRIEF)-P (13). Details of the developmental tests have been previously described (13).

We determined UIC by using spectrophotometry and thyroid function, thyroglobulin and TPO-Ab were measured using immunoassays, as previously described (13). With the exception of TSH and total thyroxine during pregnancy, we used the manufacturer's reference ranges. For TSH during pregnancy, we used trimester specific reference ranges: 0.1–2.5 mIU/L for 1st trimester, 0.2–3.0 mIU/L for 2nd trimester, and 0.3–3.0 mIU/L for 3rd trimester (14). For total thyroxine until week 6, we used the reference range of 58–161 nmol/L; from week 7, we increased the upper reference range by 5% per week until week 15; from week 16 until delivery, we multiplied the non-pregnancy reference range by 1.5 and used the resulting range of 87–241.5 nmol/L as a reference (15).

In children, TSH and total thyroxine concentrations were measured on filter paper by fluoroimmunoassay (TSH, DELFIA NeoTSH, PerkinElmer Life Sciences, Turku, Finland; and T4, Delfia Neonatal T4 kit, PerkinElmer Life Sciences). We applied age-specific reference ranges for TSH and total thyroxine as supplied by the manufacturer: TSH 0.1–3.7 mIU/L and total thyroxine 65–165 nmol/L.

### Data and Statistical Analysis

As described previously (13), to discriminate a 5-point difference on the full scale IQ score of the WPPSI-III between groups at 5 and 6 years, with a standard deviation of 15, α of 0.05 (two-sided), and 80% power, we estimated we would need 142 children in each group. We used WHO criteria based on the mUIC to classify adequate iodine intake for pregnant women (≥150 μg/L) infants and children (≥100 μg/L) (4). Overt hypothyroidism was defined as a high TSH and a low total thyroxine; subclinical hypothyroidism as a high TSH and a normal total thyroxine; isolated hypothyroxinemia (in the pregnant women) was defined as a normal TSH and a low total thyroxine; overt hyperthyroidism was defined as a low TSH and a high total thyroxine; subclinical hyperthyroidism as a low TSH and a normal total thyroxine. From the thyroid ultrasound measurements, we calculated thyroid volume as previously described (4).

We conducted data analysis with the R statistical programming environment (version 3.6.1) (16) using packages nlme and lme4, as previously described (13). Data were presented as mean (±SD) for normally distributed data, median (IQR) for non-normal data, and percentage (n) for prevalence. We assessed the intervention effect by fitting linear mixed effects models to continuous dependent variables and by fitting logistic regression mixed effects models to categorical dependent variables, as described previously (13). During gestation, the fixed effects were defined as treatment, trimester, treatment by trimester, maternal education, household average monthly income, maternal age and maternal BMI at trial entry to improve the model fit. For the primary outcomes in children at 5–6 years and the BDIS-III at 1 and 2 year, we defined the fixed effects as treatment, household average monthly income, maternal education, child's birthweight, child's age and child's sex. We defined subject as the random effect. We ran a sensitivity analysis for maternal outcomes during pregnancy. The pregnant women in the iodine group were divided into three tertiles based on their mean UIC from the three trimesters. We assessed the effect of the sensitivity analysis by fitting linear mixed effect models and by fitting logistic regression mixed effect models, as previously described, with two additional fixed effects defined as tertile and tertile by time. Statistical significance was set at *P* < 0.05.

## Results

**Figure 1:**
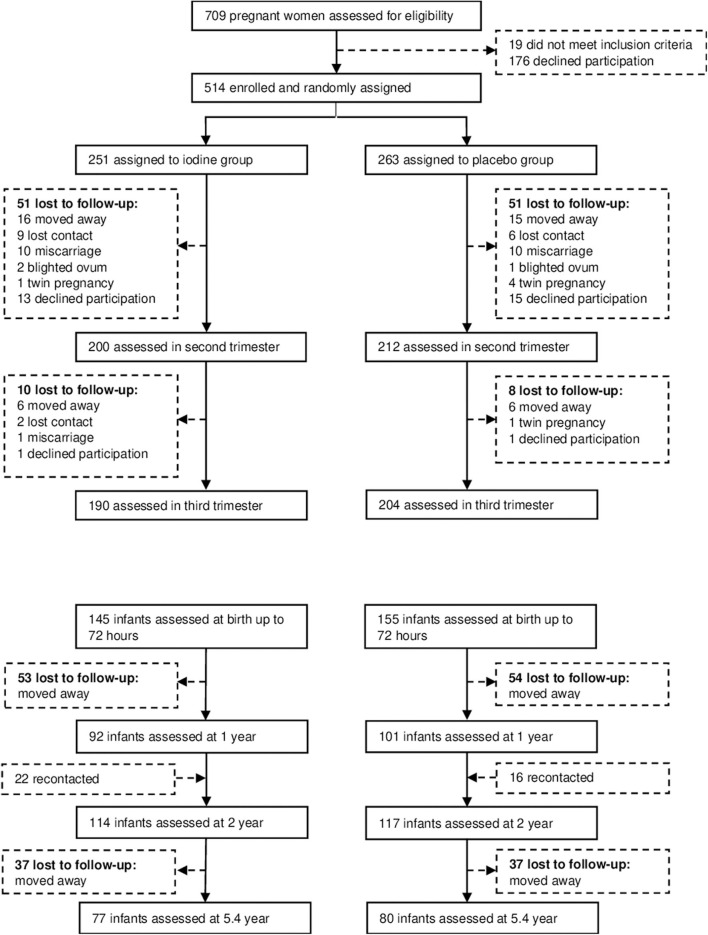
Study overview.

We recruited subjects between November 2008 and March 2011, and completed the data collection in July 2016. We assessed 709 participants for eligibility; 195 declined to participate or did not meet the inclusion criteria (Figure 1). 514 pregnant women were enrolled and randomized to the two groups. Participants' baseline characteristics are shown in Table 1. In the iodine and placebo groups, mean (±SD) gestational age at trial entry was 10.9 (2.8) and 11.0 (2.9) weeks and mUIC (IQR) was 112 (76–163) and 110 (70–172) μg/L, indicating mild iodine deficiency (4). There were no significant differences between the groups in any of the baseline variables, with the exception of a slightly higher free thyroxine in the iodine group (*p* = 0.04) (Table 1). The prevalence of mild thyroid hypofunction, that is, isolated hypothyroxinemia and/or subclinical hypothyroidism, was about 12% in both groups.

**Table 1:** Baseline characteristics of the Thai pregnant women, by group.

	**Iodine**		**Placebo**	
	***n***	**Value**	***n***	**Value**
Age^a^, y	251	30.0 (5.2)	263	29.7 (5.1)
BMI, kg/m^2^	250	21.5 (19.5–23.6)	263	21.5 (19.6–23.8)
Gestational age, wk	251	10.9 (2.8)	263	11.0 (2.9)
First pregnancy, n (%)	251	118 (47)	263	106 (40)
Using iodized salt at home, n (%)	251	222 (88)	263	231 (88)
Urinary iodine concentration^b^, μg/L	248	112 (76–163)	260	110 (70–172)
Thyroid volume, mL	251	8.58 (1.88)	263	8.72 (2.08)
Thyroid stimulating hormone, mIU/L	245	1.0 (0.6–1.7)	258	1.1 (0.7–1.7)
Thyroglobulin, μg/L	232	9.38 (4.65–16.53)	239	9.57 (6.36–16.85)
Free T_3_, ng/L	237	3.13 (2.70–3.58)	252	3.21 (2.78–3.68)
Total T_3_, nmol/L	248	1.85 (1.56–2.15)	262	1.85 (1.54–2.19)
Free T_4_, ng/L	240	1.08 (0.98–1.20)	258	1.07 (0.95–1.18)
Total T_4_, nmol/L	247	118 (101–138)	259	117 (102–135)
TPO-Ab, IU/mL	251	17.7 (11.6–24.7)	263	16.2 (11.1–26.1)
Raised TPO-Ab >35 IU/mL, n (%)	251	37 (15)	263	40 (15)
Isolated hypothyroxinaemia, n (%)	243	4 (2)	256	2 (1)
Subclinical hypothyroidism, n (%)	243	24 (10)	256	26 (10)
Overt hypothyroidism, n (%)	243	0 (0)	256	0 (0)
Subclinical hyperthyroidism, n (%)	243	10 (4)	256	11 (4)
Overt hyperthyroidism, n (%)	243	3 (1)	256	1 (<1)

a *Continuous data were analyzed using linear mixed effect models with non-transformed or transformed dependent variables and frequencies were analyzed using mixed effect logistic regression testing the differences between treatment populations (iodine or placebo)*.

b *Mean (±SD) (all such values)*.

*T_3_, tri-iodothyronine; T_4_, thyroxine; TPO-Ab, antithyroid peroxidase antibodies*.

Mean compliance with supplementation was 88%. Table 2 shows the maternal variables during pregnancy, by group. We analyzed data from 412 women in the 2nd trimester and 394 women in the 3rd trimester. In the 2nd and 3rd trimesters, mUIC in the iodine group was 224 and 233 μg/L, indicating clear iodine sufficiency in the iodine group (4), while in the placebo group, it was 144 and 155 μg/L, suggesting borderline iodine sufficiency (*p* < 0.001). During pregnancy, maternal free and total thyroxine concentrations were lower in the iodine group as compared to placebo (*p* = 0.0003, and *p* = 0.02, respectively). In a sensitivity analysis based on UIC tertiles during pregnancy restricted to the iodine group, we saw a steeper decline in free and total thyroxine concentrations in women over increasing tertiles of UIC, but these trends were not statistically significant. As expected, maternal thyroglobulin concentrations were lower in the iodine group (*p* = 0.0006). There were no significant group differences in any thyroid disorders (Table 2). The analysis were conducted with and without gestational age as fixed factor, but the results did not change for any of the parameters (data not shown).

**Table 2:** Maternal outcomes in Thai pregnant women, by group.

	**Baseline**		**Second trimester**		**Third trimester**		
	***n***	**Value**	***n***	**Value**	***n***	**Value**	***P*^a^**
Urinary iodine concentration^b^, μg/L							
Iodine	248	112 (76–163)	167	224 (133–345)	171	233 (140–313)	<0.001
Placebo	260	110 (70–172)	152	144 (86–237)	185	155 (108–211)	
Thyroid volume^c^, mL							
Iodine	251	8.58 (1.88)	185	8.44 (1.92)	176	8.17 (2.05)	0.59
Placebo	263	8.72 (2.08)	171	8.54 (2.25)	190	8.20 (1.93)	
Thyroid stimulating hormone, mIU/L							
Iodine	245	1.0 (0.6–1.7)	173	1.2 (0.8–1.8)	156	1.1 (0.7–1.7)	0.13
Placebo	258	1.1 (0.7–1.7)	165	1.2 (0.8–1.7)	177	1.3 (0.8–1.8)	
Thyroglobulin, μg/L							
Iodine	232	9.38 (4.65–16.53)	167	8.60 (4.65–15.45)	151	9.78 (5.50–18.10)	<0.001
Placebo	239	9.57 (6.36–16.85)	151	10.4 (5.65–15.80)	175	11.9 (6.48–22.25)	
Free tri-iodothyronine, ng/mL							
Iodine	237	3.13 (2.70–3.58)	162	3.35 (2.82–3.99)	150	3.32 (2.96–3.83)	0.57
Placebo	252	3.21 (2.78–3.68)	149	3.22 (2.89–3.72)	175	3.38 (2.91–3.85)	
Total tri-iodothyronine, nmol/L							
Iodine	248	1.85 (1.56–2.15)	180	2.38 (2.03–2.77)	164	2.44 (2.13–2.73)	0.45
Placebo	262	1.85 (1.54–2.19)	163	2.34 (2.00–2.68)	182	2.42 (2.13–2.86)	
Free thyroxine, ng/dL							
Iodine	240	1.08 (0.98–1.20)	175	0.78 (0.72–0.88)	161	0.77 (0.68–0.84)	<0.001
Placebo	258	1.07 (0.95–1.18)	162	0.81 (0.75–0.88)	185	0.79 (0.71–0.87)	
Total thyroxine, nmol/L							
Iodine	247	118 (101–138)	175	114 (102–129)	161	113 (100–129)	0.02
Placebo	259	117 (102–135)	167	116 (102–131)	182	117 (101–131)	
TPO-Ab, IU/mL							
Iodine	251	17.7 (11.6–24.7)	183	13.7 (8.5–21.6)	170	12.8 (5.5–19.6)	0.69
Placebo	263	16.2 (11.1–26.1)	171	14.2 (9.0–21.7)	192	11.7 (6.2–18.9)	
Raised TPO-Ab >35 IU/mL, n (%)							
Iodine	251	37 (15)	183	21 (11)	170	11 (6)	0.89
Placebo	263	40 (15)	171	15 (9)	192	13 (7)	
Isolated hypothyroxinaemia, n (%)							
Iodine	243	4 (2)	170	19 (11)	155	13 (8)	0.98
Placebo	256	2 (1)	164	11 (7)	176	14 (8)	
Subclinical hypothyroidism, n (%)							
Iodine	243	24 (10)	170	11 (6)	155	6 (4)	0.57
Placebo	256	26 (10)	164	6 (4)	176	4 (2)	
Overt hypothyroidism, n (%)							
Iodine	243	0 (0)	170	2 (1)	155	1 (1)	0.16
Placebo	256	0 (0)	164	0 (0)	176	0 (0)	
Subclinical hyperthyroidism, n (%)							
Iodine	243	10 (4)	170	4 (2)	155	7 (5)	0.51
Placebo	256	11 (4)	164	4 (2)	176	4 (2)	
Overt hyperthyroidism, n (%)							
Iodine	243	3 (1)	170	0 (0)	155	0 (0)	0.25
Placebo	256	1 (<1)	164	0 (0)	176	0 (0)	
Sum of thyroid disorders^d^, n(%)							
Iodine	251	41 (16)	183	36 (20)	170	27 (16)	0.78
Placebo	263	40 (15)	171	21 (12)	192	22 (11)	

a *Continuous data were analyzed using linear mixed effect models with non-transformed or transformed dependent variables. Frequencies were analyzed using mixed effect logistic regression. The interaction effect of treatment by time was tested while controlling for household average monthly income, maternal education, maternal age and maternal BMI at trial entry*.

b *Median (IQR) (all such values)*.

c *Mean (±SD) (all such values)*.

d *Sum of the following disorders: isolated hypothyroxinaemia, subclinical hypothyroidism, overt hypothyroidism, subclinical hyperthyroidism, and overt hyperthyroidism*.

*TPO-Ab, antithyroid peroxidase antibodies*.

We analyzed data from 193 infants at 1 year, 231 infants at age 2 y and 157 children at mean age 5.7 y; attrition was balanced between groups and the main reason was a move away from the trial area around delivery (Figure 1). At age 2 years, the TSH concentrations were lower in the iodine group (*p* = 0.03). We did not observe significant group differences in anthropometrics, UIC and total thyroxine in infants at age 1, 2, and 5.7 years (Table 3). At age 1 year, there were no significant group differences on the BSID-III (Table 4). At age 2 years, the iodine group had lower scores for gross motor (*p* = 0.07) and combined fine and gross motor function (*p* = 0.05) with mean differences [95% CI] of −0.8 [−1.6 to 0.03] and −1.1 [−2.2 to 0.03], respectively (Table 4). This finding was in part driven by the low motor scores of two children; after removing these two cases from the analysis, the differences in the gross motor and combined motor function scores were reduced (mean diff=-0.6, *p* = 0.14 and mean diff=-0.8, *p* = 0.12, respectively). At 5.7 years, there were no significant group differences on the WPPSI-III or the BRIEF-P (Table 5). However, on all four measured categories of the WPPSI-III, verbal IQ, performance IQ, processing speed and full scale IQ, the iodine group had lower scores with mean differences between 0.2 and 2.0 points, although these differences were not statistically significant. As discussed below, a limitation of this secondary analysis is that the sample size at 5.7 years (*n* = 157) was below the estimated sample size needed to discriminate a 5-point difference on the WPPSI (13). All adverse events during gestation and delivery were recorded; there was no significant group difference in frequency of adverse events: 14 women in the iodine group and 16 in the placebo group experienced adverse events.

**Table 3:** Secondary outcomes in Thai children, at age 1, 2, and 5.7 years, by group.

	**1 year**			**2 year**			**5.7 year**		
	***n***	**Value**	***P*^a^**	***n***	**Value**	***P*^a^**	***n***	**Value**	***P*^a^**
Sex, male, n (%)									
Iodine	92	38 (41)	0.43	115	50 (43)	0.56	77	32 (42)	0.84
Placebo	104	49 (47)		118	55 (47)		80	35 (44)	
Age^b^, years									
Iodine	92	1.0 (0.1)	0.20	114	2.1 (0.2)	0.97	77	5.6 (0.6)	0.36
Placebo	101	1.0 (0.1)		117	2.1 (0.2)		80	5.7 (0.6)	
Bodyweight, kg									
Iodine	92	9.3 (1.2)	0.94	113	12.3 (2.0)	0.98	77	19.7 (4.6)	0.08
Placebo	101	9.3 (1.2)		116	12.3 (2.0)		80	21.0 (6.2)	
Height, cm
Iodine	92	75.0 (3.5)	0.21	114	87.0 (3.6)	0.74	77	112.1 (6.0)	0.32
Placebo	101	74.6 (3.0)		117	86.9 (4.0)		80	113.2 (6.4)	
Height-for-age Z score									
Iodine	91	−0.1 (1.3)	0.20	114	−0.2 (1.1)	0.69	77	−0.2 (1.0)	0.34
Placebo	100	−0.3 (1.1)		117	−0.3 (1.1)		80	−0.1 (1.0)	
Stunting, n (%)									
Iodine	91	5 (5)	0.53	114	6 (5)	0.77	77	2 (3)	0.17
Placebo	100	4 (4)		117	7 (6)		80	0 (0)	
Urinary iodine concentration^c^, μg/L									
Iodine	58	239 (136–386)	0.29	82	231 (149–293)	0.62	77	243 (154–350)	0.70
Placebo	64	273 (184–438)		86	223 (160–325)		78	260 (180–311)	
Total thyroxine, nmol/L									
Iodine	75	58.5 (41.6–80.9)	0.39	103	64.0 (49.3–83.3)	0.42	76	80.0 (70.1–91.0)	0.11
Placebo	86	60.8 (45.4–88.3)		107	62.6 (47.7–79.8)		77	83.3 (69.1–99.7)	
Thyroid stimulating hormone, mIU/L									
Iodine	75	0.7 (0.6–1.0)	0.11	103	0.8 (0.6–1.0)	0.03	76	0.9 (0.7–1.1)	0.12
Placebo	86	0.8 (0.6–1.0)		107	0.9 (0.6–1.1)		77	0.9 (0.7–1.2)	

a *Continuous data were analyzed using linear mixed effect models with non-transformed or transformed dependent variables. Frequencies were analyzed using mixed effect logistic regression. The interaction effect of treatment by time was tested while controlling for household average monthly income, maternal education, child's birthweight, child's age and child's sex*.

b *Mean (±SD) (all such values)*.

c *Median (IQR) (all such values)*.

**Table 4:** Cognitive outcomes in Thai children, at age 1 and, 2 years, by group.

	***n***	**1 year**	**Mean difference (95% CI)**	***P***	***n***	**2 year**	**Mean difference (95% CI)**	***P*^a^**
BSID-III, Cognitive^b^								
Iodine	85	42 (40–45)	1.1 (0.01 to 2.3)	0.08	108	62 (59–64)	−0.2 (−1.4 to 0.9)	0.52
Placebo	92	42 (40–43)			110	62 (59–65)		
BSID-III, Language receptive								
Iodine	85	12 (11–13)	0.4 (−0.04 to 0.8)	0.18	107	26 (24–29)	0.5 (−0.7 to 1.7)	0.60
Placebo	90	12 (11–12)			108	25 (23–28)		
BSID-III, Language expressive								
Iodine	85	13 (11–14)	−0.05 (−0.8 to 0.7)	0.65	104	29 (26–31)	0.9 (−0.4 to 2.3)	0.38
Placebo	90	13 (12–15)			104	29 (26–31)		
BSID-III, Language combined								
Iodine	85	25 (23–28)	0.3 (−0.6 to 1.3)	0.79	104	55 (50–59)	1.5 (−0.8 to 3.8)	0.40
Placebo	90	25 (23–27)			104	54 (49–58)		
BSID-III, Fine motor								
Iodine	85	29 (28–30)	0.3 (−0.3 to 0.9)	0.41	106	37 (36–39)	−0.3 (−0.9 to 0.4)	0.37
Placebo	90	29 (27–30)			108	38 (36–39)		
BSID-III, Gross motor								
Iodine	84	41 (38–44)	−0.2 (−1.5 to 1.0)	0.39	104	54 (52–55)	−0.8 (−1.6 to 0.03)	0.07
Placebo	90	41 (38–44)			107	54 (52–56)		
BSID-III, Motor combined								
Iodine	84	70 (66–73)	0.1 (−1.4 to 1.6)	0.76	103	91 (89–93)	−1.1 (−2.2 to 0.03)	0.05
Placebo	90	70 (67–73)			107	92 (90–94)		

a *Data were analyzed using linear mixed effect models testing treatment effect while controlling for household average monthly income, maternal education, child's birthweight, child's age and child's sex*.

b *Median (IQR) all such values*.

*BSID-III, Bayley Scales of Infant Development Third Edition*.

**Table 5:** Cognitive outcomes in Thai children, at mean age 5.7 years, by group.

	***n***	**5.7 years**	**Mean difference (95% CI)**	***P^a^***
WPPSI-III, verbal IQ^b^				
Iodine	77	90.9 (± 9.3)	−0.5 (−3.5 to 2.6)	0.78
Placebo	79	91.3 (± 10.2)		
WPPSI-III, performance IQ				
Iodine	77	101.7 (± 12.9)	−2.0 (−6.2 to 2.3)	0.42
Placebo	79	103.7 (± 14.3)		
WPPSI-III, processing speed				
Iodine	77	114.3 (± 12.4)	−0.2 (−4.2 to 3.8)	0.75
Placebo	79	114.5 (± 13.0)		
WPPSI-III, full scale IQ				
Iodine	77	97.5 (± 10.4)	−1.0 (−4.4 to 2.3)	0.55
Placebo	79	98.5 (± 11.1)		
BRIEF-P, global executive				
Iodine	77	112.8 (± 19.1)	0 (−6.0 to 6.0)	0.98
Placebo	79	112.7 (± 19.2)		

a *Data were analyzed using linear mixed effect models testing treatment effect while controlling for household average monthly income, maternal education, child's birthweight, child's age and child's sex*.

b *Mean (±SD) (all such values)*.

*BRIEF-P, Behavior Rating Inventory of Executive Function Preschool Version; IQ, intelligence quotient; WPPSI-III, Wechsler Preschool and Primary Scale of Intelligence Third Edition*.

## Discussion

This secondary analysis of our previous RCT provides new insights into the effects of iodine supplementation in mildly iodine deficient women. The Thai pregnant women included in this secondary analysis at baseline had a mUIC of 112 μg/L; this degree of mild maternal iodine deficiency is common worldwide: in national studies of pregnant women in Europe, ≈2/3rds reported mild iodine deficiency based on the mUIC (17). The 200 μg dose of iodine given in this study was effective: in the iodine group, mUIC increased to 224–233 μg/L in the 2nd and 3rd trimesters, at the upper end of mUIC reference range for pregnancy of 150–249 μg/L (4). However, the mUIC in the placebo group also increased to a lesser degree (Table 2); this may have been due to a physiological increase in renal iodine clearance (2) and/or to increased awareness of iodine nutrition and consumption of more iodine-rich foods.

Iodine supplementation was associated with a small but significant negative effect on maternal thyroxine concentrations. Although there was no significant effect on TSH concentrations, free and total thyroxine concentrations declined slightly but significantly more in the iodine group over the duration of pregnancy, as reported before with a different statistical approach (18). The small observed differences in maternal thyroxine concentrations may not be of clinical significance, as there were no significant group differences in maternal hypothyroidism or isolated hypothyroxinemia between groups (Table 2). In seven previous RCTs of maternal iodine supplementation looking at maternal thyroid function (19–26), there were no significant differences in maternal TSH between the iodine and control groups. Six found no significant difference in maternal free (19, 21–24) or total thyroxine concentration (25) between the iodine and control groups, while one found a slightly higher total thyroxine concentration (26) in the iodine group. Another recent intervention study comparing daily administration of a multiple micronutrient supplement containing 300 μg or an iron and folic acid supplement in moderately iodine deficient women (mUIC 51 μg/L (IQR 33–82) found no differences in maternal TSH or total thyroxine concentration (27). Other intervention studies (that were not RCTs) found no difference in free thyroxine concentrations comparing different doses of iodine supplements (28, 29), but one reported a lower free thyroxine concentration in women receiving iodine supplements (30). One study reported that women who started taking 150 μg iodine/day (as part of a multivitamin/mineral tablet) had a significantly lower free thyroxine concentrations during pregnancy than women who started using iodized salt before pregnancy (31). Most cross-sectional studies associating iodine supplements with maternal thyroxine concentrations found no difference in free thyroxine (32–34) or total thyroxine concentrations (35), although two studies reported lower free thyroxine (36, 37) in iodine-supplement users. A large cross-sectional Chinese study (in an iodine-sufficient region) reported higher mUICs during early pregnancy were associated with mild maternal hypothyroidism (9): among pregnant women (*n* = 7,190) at 4–8 wk gestation, spot UICs of 250–499 μg/L and ≥500 μg/L were associated with a 1.7-fold and a 2.2-fold increased risk of subclinical hypothyroidism, respectively, and the latter UIC with a 2.9-fold increased risk of isolated hypothyroxinemia (9). Our study suggests that during mild maternal iodine deficiency, thyroid adaptation maintains maternal thyroxine concentrations within the normal range, and although there may be a small risk of decreased thyroxine concentrations during iodine supplementation, the data remain uncertain.

Notably, we found borderline non-significant group differences in child motor outcomes at age 2 years (Table 4). In the Spanish Infancia y Medio Ambiente–Environment and Childhood (INMA) Study cohort, in Valencia (*n* = 691), maternal supplemental iodine intake of ≥150 μg/day, compared with <100 μg/day, was associated with a significant 5.2-point decrease in psychomotor scores and a 1.8-fold increase in the odds of a psychomotor score <85 at age 1 year (10). When pooled with the three other sites in INMA (*n* = 1,519), the overall increase in risk for a psychomotor score <85 associated with maternal supplemental iodine intake of ≥150 μg/day remained significant (OR = 1.7, 95% CI: 1.1, 2.6) (11). When stratified by maternal iodized salt consumption, this increase in risk was greater for women who consumed iodized salt, suggesting that the extra dose of iodine from supplements might result in iodine excess (11). However, these negative effects on child development in the INMA cohort were not confirmed at age 4–5 years (38). Similarly, we found no significant differences in cognitive outcomes at age 5.7 years, but on all four measured categories of the WPPSI-III, the iodine group had lower scores, with mean differences between 0.2 and 2.0 points (Table 5). In an observational study from Norway (*n* = 851, mUIC=78 μg/L), maternal use of iodine-containing supplements (150–200 μg/d) during pregnancy predicted poorer fine motor skills (standardized beta = −0.10, *p* = 0.15) and gross motor skills (standardized beta = −0.18, *p* = 0.02), measured using the BSID-III at 18 months (12).

Iodine readily crosses the placenta and excessive iodine intake during pregnancy can cause iodine-induced hypothyroidism in the fetus and newborn (39). This phenomenon is known as the acute Wolff-Chaikoff effect and in adults, an “escape” from the acute Wolff-Chaikoff effect usually occurs after a few days of exposure. However, the immature fetal thyroid gland is unable to escape from the acute Wolff-Chaikoff effect, making the fetus and newborn vulnerable to iodine-induced hypothyroidism (39–41). Fetal hypothyroidism may develop in the setting of a large iodine load even if maternal thyroid function remains normal (39). Although most reports of fetal and newborn iodine-induced hypothyroidism have occurred after very large maternal doses of iodine (typically in the mg range of intake), it is possible that lower supplemental doses could cause this effect in susceptible pregnancies. Therefore, iodine intake during pregnancy should be high enough to ensure normal thyroid function in the mother and fetus, but more iodine is not necessarily better.

This secondary analysis has several limitations. The women began iodine supplementation at mean gestational age of 11 wk. It is possible that beginning supplementation earlier would have resulted in different findings, as the fetal brain rapidly develops in the 1st trimester (2, 3). However, we did not find an effect of gestational age at entry on any of the developmental outcomes in secondary analysis of the iodine group. We used the mUIC from spot samples to classify population iodine status in pregnant women per recommendations from WHO, but UIC is a poor marker of individual status (4). Thus, it is uncertain whether all women included in this study were iodine deficient and exposure misclassification may have biased our results. The dropout rate was high around delivery but attrition was balanced between groups. The final sample size at 5.7 years (*n* = 157) was below the estimated sample size (*n* = 284) needed to discriminate a 5-point difference on the WPPSI between groups; thus, the lack of a group effect on cognitive outcomes at age 5.7 years in this secondary outcome is inconclusive.

In conclusion, in this secondary analysis, daily iodine supplementation with 200 μg iodine in mildly iodine deficient pregnant women did not benefit their thyroid function or improve child development. Iodine supplementation resulted in small but significant negative effects on maternal thyroid function, but did not affect child development. Currently, the American and European Thyroid Associations (6, 7) recommend women take a supplement containing 150 μg iodine daily during pregnancy. In contrast, WHO does not recommend maternal iodine supplements in countries with iodized salt programs (42), concluding that if women are iodine-replete before they enter pregnancy, they can cover their requirements by increasing fractional clearance of plasma iodide and drawing from thyroidal iodine stores. Our findings suggest current expert recommendations for iodine supplementation in this setting need to be evaluated in large randomized controlled trials that assess both safety and efficacy. Also, it would be valuable for future intervention trials to include pregnant women with moderate iodine deficiency (e.g., with a mUIC <100 μg/L).

## Data Availability Statement

The data analyzed in this study is subject to the following licenses/restrictions: Dataset can be obtained on request. Requests to access these datasets should be directed to MZ, michael.zimmermann@hest.ethz.ch.

## Ethics Statement

Ethical review boards at Ramathibodi Hospital and at Wageningen University & Research, the Netherlands, approved the study. All participating women gave written informed consent.

## Author Contributions

SG, AM-B, PW, and MZ designed the study. SG, PW, and AM-B did the field work. SG did the laboratory analyses. NV and MA did the statistical analysis. MZ wrote the first draft of the report. All authors edited and approved the final text.

## Conflict of Interest

The authors declare that the research was conducted in the absence of any commercial or financial relationships that could be construed as a potential conflict of interest.
